# Negative factors of personality hardiness that effect on ability to control situation and cope with the stress

**DOI:** 10.1192/j.eurpsy.2024.1725

**Published:** 2024-08-27

**Authors:** S. Tukaiev, D. Kashpur, N. Pogorilska, M. Makarchuk, I. Zyma, J. M. A. Ferreira

**Affiliations:** ^1^Faculty of Communication, Culture, and Society, Institute of Public Health, Università della Svizzera italiana, Lugano, Switzerland; ^2^ Institute of Biology and Medicine; ^3^Faculty of Psychology, National Taras Shevchenko University of Kyiv; ^4^Research Institute, National University of Ukraine on Physical Education and Sport, Kyiv, Ukraine; ^5^Faculdade de Medicina, Universidade de Coimbra, Coimbra, Portugal

## Abstract

**Introduction:**

Personality hardiness expresses the characteristics that help to overcome stress and achieve well-being.

**Objectives:**

This study focused on the Hardiness as the important personality trait, which allow coping with stress and the relationship of empathy, emotional sensitivity and the personality hardiness.

**Methods:**

88 healthy volunteers, students aged 17 to 26 years (mean age = 19, SD = 1,69), participated in this study. We used Cloninger’s Temperament and Character Inventory (TCI), the Maddi Hardiness Survey (adapted by Leontyev), Buss Perry Aggression Questionnaire (BPAQ), the Barratt impulsiveness scale (BIS-11), Maslach Burnout Inventory (MBI), the Questionnaire Measure of Emotional Empathy (QMEE).

**Results:**

The cluster analysis was used to identify groups of hardy personalities. We demonstrated a negative relationship between hardiness and depression and burnout. It revealed significant differences between these groups by the following traits: Attention (BIS-11), Self-Control (BIS-11), Cognitive Complexity (BIS-11), Hostility (BPAQ), Exploratory activity (NS1 TCI), Shyness of strangers (HA3 TCI), Resourcefulness (S3 TCI). Regression analysis was used to identify Hardiness factors and to build the following regression models. For the first group the models describe 100% of dispersion (R-square=1,000, Durbin-Watson statistic = 1,419) and are:

Control = -16,998 - 2,922*С2 + 3,549*С5 + 3,264*CI + 0,723*ST2 + 0,747*S4 - 0,306*SC + 0,166*RD3 - 0,020*C — 0,003*NS2, where *C2* – scale Empathy (TCI), *C5* – scale Principles (TCI), *CI* – cognitive instability, *ST2* – Transpersonal identification scale (TCI), *S4* – Self-acceptance (TCI), *SC* – Self-Control (BIS-11), *RD3* – Social attachment (TCI), *C* – Cooperativeness (TCI), *NS2* – Impulsive decision making (TCI).

The *Hardiness* model described 50% (R-square=0,456) of dispersion:

Hardiness = 63,527 – 4,080*C2, where *C2* –Empathy scale (TCI) (*p=0,003*).

The regression models of the second group explain 50% of group dispersion (R-square=0,512) and are Independent variables significance *p<0,05*:

Challenge = 12,484 + 0,389*SC + 0,197*EE — 0,702*RD1 — 0,206*A,where *SC*- Self-Control scale (BIS-11), *EE* – Emotional Empathy (Personality test of Emotional Empathy), *RD1* – Sentimentality scale (TCI), *A –* Anger (BPAQ).

The *Hardiness* model describes 35% of dispersion (R-square=0,364, Durbin-Watson statistic = 2,066):

Hardiness = 100,352 + 0,941*SC — 0,527*H, where *SC –* Self-Control scale (BIS-11) (*p=0,009*), *H* – Hostility scale (BPAQ) (*p=0,021*).

**Conclusions:**

Thus, the attention and self-control problems, hostility, cognitive complexity and shyness have a negative impact on hardiness.Our results suggest that the excessive use of empathy leads to decrease of ability to control situation and cope with the stress.

**Disclosure of Interest:**

None Declared

